# Aquaporin 4 deletion exacerbates brain impairments in a mouse model of chronic sleep disruption

**DOI:** 10.1111/cns.13194

**Published:** 2019-07-31

**Authors:** Rui Zhang, Yun Liu, Yan Chen, Qian Li, Charles Marshall, Ting Wu, Gang Hu, Ming Xiao

**Affiliations:** ^1^ Department of Neurology the First Affiliated Hospital of Nanjing Medical University Nanjing China; ^2^ Jiangsu Province, Key Laboratory of Neurodegeneration Nanjing Medical University Nanjing China; ^3^ Brain Institute the Affiliated Nanjing Brain Hospital of Nanjing Medical University Nanjing China; ^4^ Department of Rehabilitation Sciences University of Kentucky Center of Excellence in Rural Health Hazard KY USA

**Keywords:** aquaporin‐4, behavioral test, glymphatic system, hippocampus, sleep disruption

## Abstract

**Aims:**

As a normal physiological process, sleep has recently been shown to facilitate clearance of macromolecular metabolic wastes from the brain via the glymphatic system. The aim of the present study was to investigate pathophysiological roles of astroglial aquaporin 4 (AQP4), a functional regulator of glymphatic clearance, in a mouse model of chronic sleep disruption (SD).

**Methods:**

Adult AQP4 null mice and wild‐type (WT) mice were given 7 days of SD using the improved rotating rod method, and then received behavioral, neuropathological, and neurochemical analyses.

**Results:**

Aquaporin 4 deletion resulted in an impairment of glymphatic transport and accumulation of β‐amyloid and Tau proteins in the brain following SD. AQP4 null SD mice exhibited severe activation of microglia, neuroinflammation, and synaptic protein loss in the hippocampus, as well as decreased working memory, compared with WT‐SD mice.

**Conclusion:**

These results demonstrate that AQP4‐mediated glymphatic clearance ameliorates brain impairments caused by abnormal accumulation of metabolic wastes following chronic SD, thus serving as a potential target for sleep‐related disorders.

## 1 INTRODUCTION

Sleep is a vital physiological state in human life, facilitating strength restoration, synaptic plasticity, and memory consolidation.^1^ Sleep insufficiency, or circadian disruption, is associated with increased risks of various disorders, such as cardiovascular diseases, Alzheimer's disease (AD), Parkinson's disease (PD), depression, and anxiety.^2^, ^3^, ^4^ Recently, sleep has been shown to facilitate clearance of metabolic wastes from the brain.^5^, ^6^ Subsequent studies indicate that the glymphatic system enhances brain macromolecular removal during the sleep period.^7^, ^8^

The glymphatic system is a brain‐wide paravascular pathway that is responsible for clearing interstitial solutes, including amyloid‐β (Aβ) and Tau proteins, from the brain parenchyma.^9^ Several groups have independently shown that fast glymphatic transport depends upon the expression and perivascular localization of the astroglial water channel aquaporin‐4 (AQP4),^10^, ^11^, ^12^ although there is literature that contradicts this view.^13^ Perivascular AQP4 supports rapid water movement between perivascular space and glial syncytium, thus forming a convective bulk flow of interstitial fluid (ISF). In turn, this promotes clearance of ISF solutes into the cerebrospinal fluid (CSF).^10^ Disrupted perivascular AQP4 polarization caused by reactive astrogliosis impairs glymphatic clearance, subsequently increasing accumulation of metabolic wastes in aged, AD and injured brains.^14^, ^15^, ^16^, ^17^, ^18^ However, the exact role of AQP4‐mediated glymphatic clearance in brain impairments following chronic sleep insufficiency remains elusive.

To address this issue, we investigated glymphatic transport and accumulation of Aβ and Tau proteins following 7 days of sleep disruption (SD) and assessed pathophysiological consequences of AQP4 deletion in this process. Our results demonstrated that AQP4 deletion exacerbated glymphatic transport dysfunction and brain impairments caused by chronic sleep insufficiency.

## 2 MATERIALS AND METHODS

### 2.1 Animals

Global Aqp4 gene knockout (KO) mice were generated on a CD1 genetic background as described previously.^19^ These animals were backcrossed for 20^+^ generations with C57BL/6N mice prior to experimentation. The present research was performed on male AQP4 null mice and wild‐type (WT) mice at 4‐5 months of age. They were randomly divided into four groups: KO‐SD, KO‐Control (Con), WT‐Con, and WT‐SD. The animal experiments were approved by the Animal Ethical and Welfare Committee of Nanjing Medical University.

### 2.2 Sleep disruption

**Figure 1:**
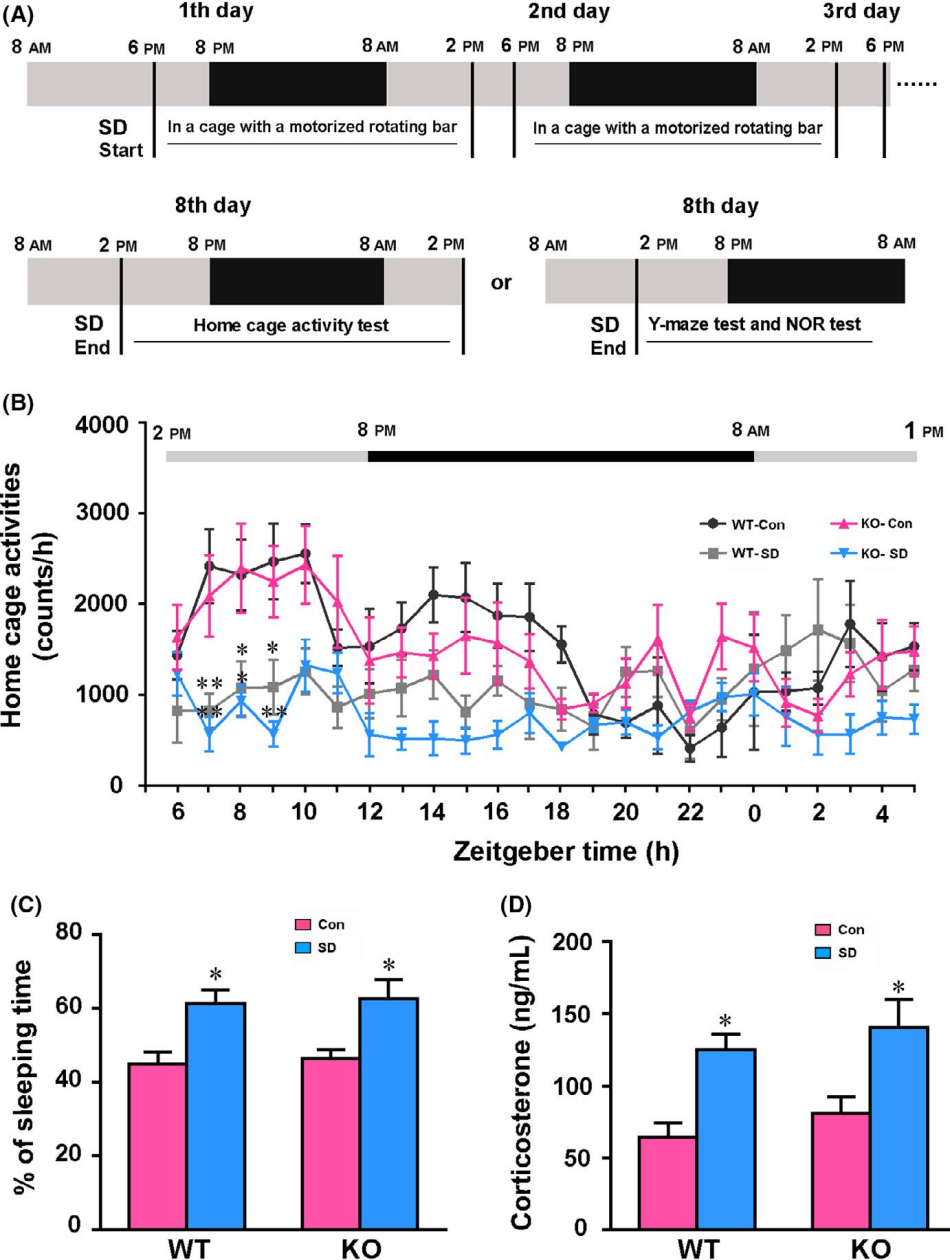
The procedures of chronic sleep disruption (SD) and analyses of rhythm and stress state. A, Diagram showing the procedures of chronic SD with behavioral tests. B, Detection of 24‐h cage activity in mice after SD. C, Percentage of sleep time during 24 h. D, Serum corticosterone concentrations of mice after SD. Under the light/dark cycle, Zeitgeber time (ZT) 0 was designated as lights on (8 am) and ZT12 (8 pm) as lights off. Data in (B) were analyzed by repeated‐measures ANOVA, and data in (C and D) were analyzed by the two‐way ANOVA with Tukey's post hoc test. Data represent mean ± SEM from eight mice per group in (B and C), and five mice per group in (D). *P < .05, **P < .01, SD vs Con

Mice were subjected to chronic SD using a modified mouse cage with a motorized rotating bar moving 10 revolutions/min.^20^, ^21^ Control mice were kept with 12:12‐hour light/dark cycles, with light on from 8 am (Zeitgeber time 0, ZT0) to 8 pm (ZT12). The procedure for sleep deprivation was performed from 6 pm (ZT10) to 2 pm (ZT6) the next day for 7 days (Figure 1A). After SD, mice received the following behavioral, neuropathological, or neurochemical analyses. All mice were housed in a controlled room temperature of 18‐22°C, 60% relative humidity, with food and water available ad libitum.

### 2.3 Home cage test

Home cage activity analysis of SD mice and control subgroups was monitored using the Mouse Home Cage Scan (version 3.00.; Clever Sys, Inc).^22^ This system allows automated quantification of continuous ambulatory activities of mice throughout the daily cycle. Mice were considered asleep if there was no motion for 80 seconds. Monitored results were retrieved as Microsoft Excel files for the whole testing period and summed up as counts per hour according to the indicated ZT. The percentage of time spent sleeping during the testing period was also calculated.

### 2.4 Y‐maze test

Mouse short‐term spatial working memory was examined using the Y‐maze test, as described previously.^23^, ^24^ During the training stage, one arm, the novel arm (NA), was blocked by an opaque plastic baffle. The mouse was allowed to explore the other two arms for 5 minutes. Two hours later, the mouse was free to move throughout all three arms. The time spent in the NA and the numbers of entries into the NA were calculated.

### 2.5 Novel object recognition test

The novel object recognition test (NORT) is used for assessing short‐term recognition memory in rodents.^25^ The mouse was placed in an open field box (45 cm long × 30 cm wide × 25 cm high), with a short, 5‐minute acclimatization period. The mouse was presented with two identical objects (cell culture flask filled with sand) for 5 minutes. Two hours later, one of the familiar objects was replaced by a new object (plastic cylindrical toys 10 cm high). Each mouse was allowed to re‐explore the familiar and novel objects for 5 minutes. The time spent by the mouse sniffing on each object within 2 cm was calculated. The recognition index was then obtained for each mouse by the following formula: (Novel object/Novel object + Familiar object).^25^

Mouse activity in the above behavioral apparatuses was collected using a digital video camera connected to a computer‐controlled system (Beijing Sunny Instruments Co. Ltd). All tests were performed by two independent experimenters who were blind to the animal genotype and treatment.

### 2.6 Injection of fluorescent tracer into the cisterna magna

Intracisternal perfusion of Texas Red‐dextran‐3 (TR‐d3, MW 3 kD; Invitrogen; Cat. # D3328) was performed as previously described.^10^, ^26^ Briefly, mice were deeply anesthetized and fixed on a stereotaxic apparatus. Following exposure of the posterior atlanto‐occipital membrane, 5 μL of TR‐d3 at 0.5 mg/mL in artificial CSF was infused into the cisterna magna through a 27‐gauge needle. The infusion was carried out at a rate of 1 µL/min. Thirty and forty‐five minutes after the start of infusion, animals were anesthetized again and the influx of fluorescent CSF tracer into the brain parenchyma was assessed.

### 2.7 Preparation of brain sections

After behavioral testing or CSF tracer injection, anesthetized mice were transcardially perfused with 50 mL 0.9% saline, followed by 60 mL 4% PFA for 5 minutes by a perfusion pump (Longer Pump, Cat # BT100‐2J). Brains were then removed and postfixed in 4% PFA at 4°C overnight. For immunohistochemical staining, postfixed brain tissues were dehydrated in a series of graded ethanol solutions and embedded in paraffin. Coronal sections containing the hippocampus were cut at 5 μm using a paraffin slicing machine (Leica RM2135). For double immunofluorescence, forebrains were dehydrated gradiently in 20% and 30% sucrose solutions for each 24 hours at 4°C. Tissue was then embedded in Tissue‐Tek OCT compound to be made into coronal sections of 30 μm thickness, using a freezing microtome (Leica CM1950). For CSF tracer experiments, PFA postfixed forebrain tissues were sliced with a vibrating microtome (World Precision Instruments Inc, WPI; Cat. # NVSLM1‐436), at a thickness of 100 μm, and mounted onto gelatin‐coated slides in sequence.

### 2.8 Immunohistochemistry

Immunohistochemical staining was performed as previously described.^18^ After deparaffinization, brain sections were incubated with mouse antiglial fibrillary acidic protein (GFAP, 1:1000; Millipore; Cat. # MAB360), rabbit anti‐ionized calcium binding adaptor molecule 1 (Iba‐1, 1:1000; Wako; Cat. # 019‐19741), or rabbit anti‐AQP4 (1:400; Millipore; Cat. # AB3594) at 4°C overnight, then with secondary antibodies labeled with horseradish peroxidase (1:200; ZSGB‐BIO; Cat. # ZB2301) at 37°C for 1 hour. Sections were visualized with 3‐3‐diaminobenzidine‐4 HCl/H_2_O_2_ (DAB Peroxidase Substrate Kit; Vector Lab; Cat. # SK‐4100).

### 2.9 Immunofluorescence

Frozen slices were blocked for 1 hour at room temperature with 5% BSA and then incubated with a mixture of primary antibodies including mouse anti‐GFAP (1:1000) and rabbit anti‐AQP4 (1:400) overnight at 4°C. Following PBS rinse, sections were incubated for 2 hours at room temperature with a mixture of Alexa Flour^™^ 555 donkey anti‐mouse IgG (1:1000; Thermo Fisher; Cat. # A31570) and Alexa Flour^™^ 488 donkey anti‐rabbit IgG (1:1000; Thermo Fisher; Cat. # A21206), followed by counterstaining with 4′, 6‐diamidino‐2‐phenylindole (DAPI, 1:1000; Thermo Fisher; Cat. # D21490) for 6 minutes and then cover‐slipped with antifluorescence quenching sealant.

### 2.10 Image analysis

All sections were visualized using a digital microscope (Leica Microsystems) and captured with constant exposure time, offset, and gain for each staining marker. The area of positive signal was measured with ImageJ (NIH) using the interest grayscale threshold analysis with constant settings for minimum and maximum intensities. The positive signal percentage area of GFAP, Iba‐1, and AQP4 was calculated, respectively, by dividing the area of positive signal to the total area in the hippocampus. For analysis of AQP4 polarization, images at 400× magnification were randomly captured from the hippocampal lacunosum molecular layer. The mean AQP4 immunofluorescent intensity of areas immediately abutting vessels and adjacent parenchyma was measured. AQP4 polarization was calculated by comparing expression ratios of AQP4 at perivascular domains vs parenchymal domains.^10^, ^18^ Three hippocampal sections per mouse, and four mice per group, were averaged to provide a mean value for each group. For the analysis of intracisternally injected TR‐d3 diffused into the brain parenchyma, the percentage area of TR‐d3‐positive signal was measured on 6 coronal sections covering +1 to −1.5 mm from anterior to posterior, relative to the bregma. Subregional analysis of CSF tracer distribution was analyzed in coronal section at the level of 0.5 mm anterior to the bregma. All quantification was done blind to animal genotype and treatment.

### 2.11 Western blotting

For immunoblotting analysis, mice were anesthetized and sacrificed by cervical dislocation and brains were quickly removed. The hippocampus was separated, then homogenized and centrifuged at 13 000 *g* for 15 minutes at 4°C in a centrifuge. The homogenized samples were loaded onto 10%‐12% Tris SDS gels, transferred onto polyvinylidene fluoride membranes, and then blocked with 5% defatted milk on a shaker for 2 hours. Following blocking, the bands were incubated at 4°C overnight with one of the following primary antibodies: amyloid beta precursor protein (APP, 1:1000; Sigma; Cat. # SAB4300464), AQP4 (1:1000; Millipore; Cat. # AB3594), apoptosis‐associated, speck‐like, caspase‐1 recruiting domain‐containing protein (ASC, 1:200; Santa Cruz; Cat. # sc‐22514‐R), Aβ_1‐40_ (1:800; Abcam; Cat. # ab12265), β‐site amyloid precursor protein‐cleaving enzyme‐1 (BACE‐1, 1:1000; Millipore; Cat. # MAB5308), GAPDH (1:3000; Bioworld; Cat. # BS60630), hyperphosphorylated Tau at Ser396/404 (PHF‐1, 1:1000; Abcam; Cat. # ab80042), IKKβ (1:1000; CST; Cat. # 8943s), insulin degrading enzyme (IDE, 1:1000; Abcam; Cat. # ab32216), interleukin‐1β (IL‐1β, 1:1000; Millipore; Cat. # AB1832P), IL‐6 (1:1000; Abcam; Cat. # ab208113), low‐density lipoprotein receptor‐related protein‐1 (LRP‐1, 1:1000; Abcam; Cat. # ab92544), neprilysin (NEP, 1:1000; Millipore; Cat. # AB5458), NOD‐like receptor protein 3 (NLRP3, 1:1000; AdipoGen; Cat. # AG‐20B‐0006), p‐IKKβ (1:1000; CST; Cat. # 2694), postsynaptic density protein 95 (PSD‐95, 1:1000; Abcam; Cat. # ab18258), presenilin1 (PS1, 1:1000; Sigma; Cat. # PRS4203), p‐P65 (1:1000; CST; Cat. # 3033s), P65 (1:1000; CST; Cat # 8242s), synaptophysin (SYP, 1:1000; Abcam; Cat. # MAB5258‐I), Tau (1:1000; Abcam; Cat. # ab32057), or tumor necrosis factor‐α (TNF‐α, 1:1000; Abcam; Cat. # ab9739). TBST washing occurred for 10 minutes three times the next day, and bands were incubated with appropriate secondary antibodies labeled with horseradish peroxidase (HRP, 1:200; ZSGB‐BIO; Cat. # ZB2301) at room temperature on a shaker for 1 hour. All bands were then visualized with enhanced chemiluminescence detection reagents (1:1; Tanon; Cat. # 180‐5001). Images were acquired through an imaging analysis system (ImageQuant^™^ LAS 4000 mini, version 1.2). Signal intensity of each band was normalized to that of GAPDH. Four mice per group, in duplicate experiments, were averaged to provide a mean value for each group.

### 2.12 ELISA measurement of brain Aβ_1‐40_ and serum corticosterone

For brain Aβ_1‐40_ ELISA analysis, hippocampal tissues were homogenized in TBS with protease inhibitors and 1% Triton X‐100. After blending, the samples were centrifuged 20 000 *g* for 30 minutes and supernatant collected and stored. The samples were assayed for quantifying Aβ_1‐40_ levels and diluted 3‐fold ahead of performing ELISA according to manufacturer's instruction.^27^ Serum corticosterone (CORT) concentration was detected using a CORT ELISA kit (R&D Systems; Cat. # KGE009).^28^ Reagents and standards were prepared according to the manufacturer's directions. Five mice per group, in duplicate experiments, were averaged to provide a mean value for each group.

### 2.13 Statistical analysis

All statistical analyses were performed using SPSS software, version 22.0 (SPSS Inc). TR‐d3 diffusion data in brain sections at different anterior‐posterior coordinates, as well as home cage activities, were analyzed by repeated‐measures ANOVA. When the assumption of sphericity was violated, a Greenhouse‐Geisser correction was used to adjust the degrees of freedom. Pearson correlation analysis was performed to evaluate the link between glymphatic dysfunction and short‐term memory deficits after chronic SD. The other data were analyzed by two‐way ANOVA with Tukey's post hoc test or Student's *t* test. *P* < .05 was considered to have statistical significance.

## 3 RESULTS

### 3.1 Chronic SD alters sleep‐wake cycle and results in stress

We assessed the consequences of 7‐day SD on mouse sleep‐wake cycles using the home cage test. Both AQP4 KO mice and WT mice subjected to SD showed significantly lower ambulatory activities between 3:00 and 7:00 pm (ZT7‐ZT11; *F*_3,166_ = 22.860, *P* < .001), the period of greatest spontaneous activities in control mice (Figure 1B). AQP4 gene deletion did not significantly affect 24‐hour activity rhythm under basic conditions (*F*_23,337_ = 4.095, *P* = .741) or after chronic SD (*F*_23,337_ = .928, *P* = .559). SD resulted in higher percentages of sleep time during the testing period in the both genotype mice (*P* < .05, WT‐SD vs WT‐Con; *P* < .05, KO‐SD vs KO‐Con; Figure 1C). Previous studies reported that sleep deprivation leads to stress response in mice with high serum CORT levels.^29^ Consistently, chronic SD increased serum CORT levels (*F*_1,16_ = 19.220, *P* = .004), but genotype effects were not obvious (*F*_1,16_ = 1.389, *P* = .256; Figure 1D).

### 3.2 AQP4 deletion aggravates impairment of glymphatic transport and accumulation of macromolecules in the brain after chronic SD

**Figure 2:**
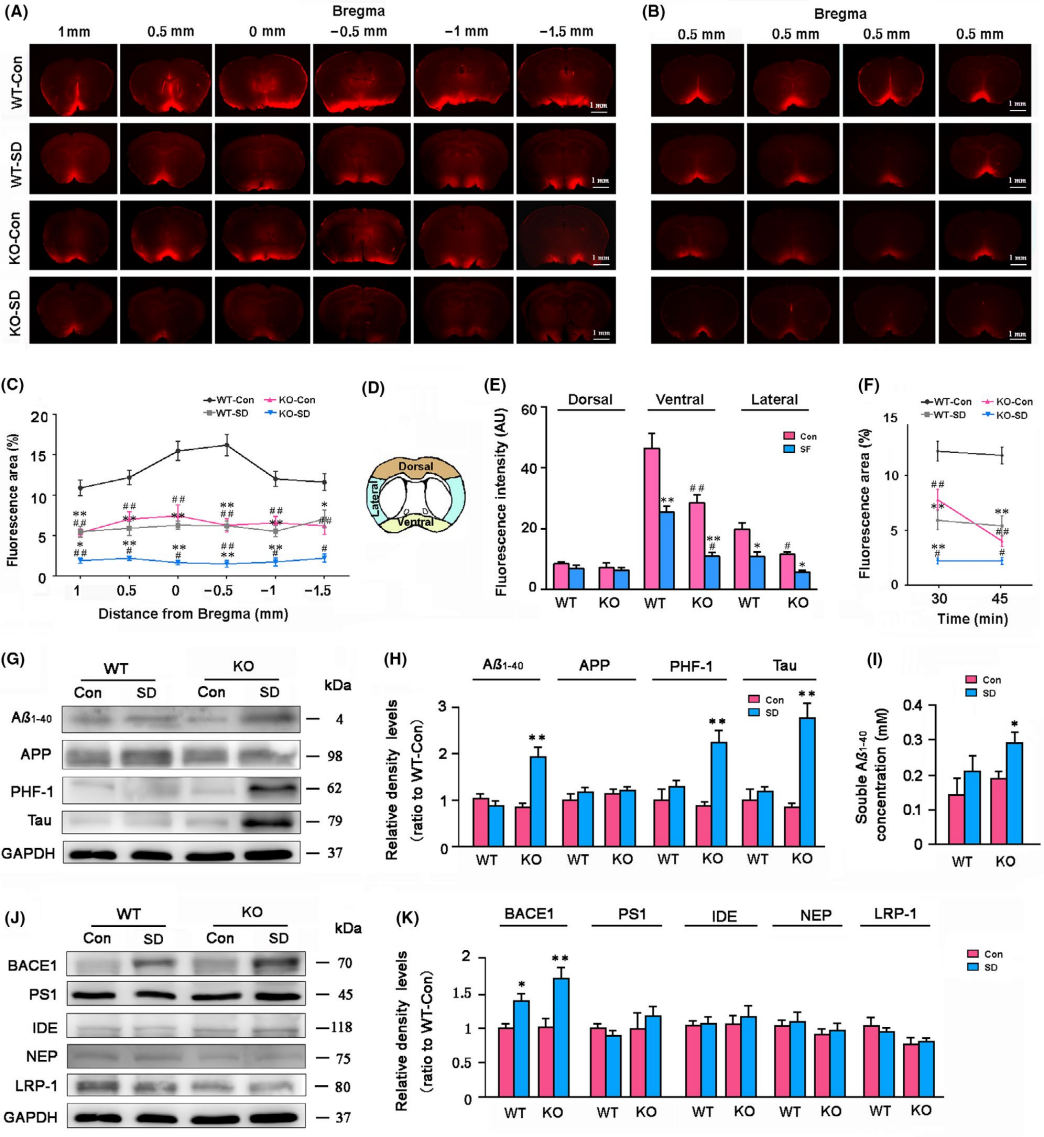
Analysis of glymphatic transport and brain macromolecule levels of WT and AQP4 KO mice following sleep disruption (SD). A, Representative images showing TR‐d3 fluorescence in six coronal brain sections from bregma +1.0 mm to −1.5 mm at 30 min after the start of injection into the cisterna magna. B, Representative images of coronal slices at bregma +0.5 mm from four mice per group showing TR‐d3 distribution within the brain parenchyma at the time point of 45 min. C, Quantification of the percentage area of TR‐d3 fluorescence in whole brain section of four groups. D, Diagram showing the subregional analysis of brain sections at the levels of bregma +0.5 mm. E, Quantification of the TR‐d3 fluorescence intensity (AU, arbitrary units) of the dorsal, ventral, and lateral brain regions at 30‐min time point, respectively. F, Quantification of the percentage area of TR‐d3 fluorescence in the coronal brain section at bregma anterior to 0.5 mm, at 30 and 45 min after the start of injection. G, H, Representative Western blot bands and densitometry analysis of the expression levels of hippocampal Aβ_1‐40_, APP, total Tau, and hyperphosphorylated Tau at Ser396/404 that are measured with PHF‐1 antibodies. I, ELISA analysis of soluble Aβ_1‐40_ levels from the hippocampal samples. J, K, Representative Western blot bands and densitometry analysis of the expression levels of Aβ generation, clearance, and transport‐related markers from the hippocampal samples. Data in (C) were analyzed by repeated‐measures ANOVA and in the other figures were analyzed by the two‐way ANOVA, with Tukey's post hoc test. Data represent mean ± SEM from four mice per group. *P < .05, **P < .01, SD vs Con; ^#^ *P* < .05, ^##^ *P* < .01, AQP4 KO vs WT. AQP4, aquaporin 4; KO, knockout; WT, wild‐type

Previous studies suggest that AQP4 is necessary for glymphatic system‐mediated ISF bulk flow.^10^ Sleep improves the influx of fluorescent CSF tracer into the brain parenchyma via the glymphatic system,^5^ while sleep deprivation suppresses glymphatic clearance.^7^, ^8^ Therefore, we evaluated the interaction between AQP4 absence and chronic SD on glymphatic transport via injection of fluorescent tracer TR‐d3 into the cisterna magna of mice. At 30 minutes after the start of the injection, glymphatic influx of TR‐d3 was globally decreased in serial sections at different anterior‐posterior coordinates after chronic SD (*F*_3,72_ = 185.300, *P* < .001), and decreases were more evident in the AQP4 KO‐SD mice than WT‐SD mice (*F*_1,36_ = 144.300, *P* < .001; Figure 2A,C). Subregional quantification on the coronal section at the level of 0.5 mm anterior to the bregma showed that CSF tracer penetrated less into the ventral and lateral brain parenchyma of AQP4 KO‐SD mice than WT‐SD mice (both *P* < .05), but was comparable in the dorsal surface of the brain, suggesting regional variables of CSF tracer influx (Figure 2D,E). Exacerbated suppression of CSF tracer influx following SD was still observed in AQP4 KO‐SD mice at 45 minutes after the start of the injection (*P* < .05, KO‐SD vs WT‐SD; Figure 2B,F). Recent studies have reported that sleep drives clearance of brain metabolites.^5^, ^6^ In contrast, sleep deprivation causes Aβ accumulation in the brain and CSF.^30^, ^31^ We determined whether AQP4 deletion aggravates impaired Aβ clearance from the brain after chronic SD. Both Western blot and ELISA revealed that SD increased Aβ_1‐40_ expression and concentrations in the hippocampus of AQP4 KO mice (*P* < .01, *P* < .05, respectively), but not in WT mice (Figure 2G‐I). Aβ_1‐40_ levels in the hippocampus were no different between AQP4 KO mice and WT mice under the basic condition. However, the levels of APP, a transmembrane protein, were not affected by SD (*F*_1,12_ = 1.079, *P* = .329) or AQP4 deletion (*F*_1,12_ = .458, *P* = .518; Figure 2G,H). Apart from Aβ_1‐40_, AQP4 contributes to glymphatic clearance of Tau from the brain.^17^ Western blot analysis revealed that SD markedly increased both hyperphosphorylated Tau at Ser396/404 (PHF‐1) and total Tau levels in AQP4 KO mice (both *P* < .01), but not in WT mice (Figure 2G,H).

We further determined whether increased hippocampal Aβ levels are also associated with altered expression levels of marker‐related Aβ generation, degradation, and transport across the brain‐blood barrier (BBB). The results demonstrate that expression levels of hippocampal BACE1, an amyloidogenic secretase, were increased by SD (*F*_1,12_ = .252, *P* = .002) but not AQP4 deletion (*F*_1,12_ = .021, *P* = .885). PS1, a key secretase in both amyloidogenic and nonamyloidogenic pathways,^32^ was not affected by either SD (*F*_1,12_ = .011, *P* = .919) or AQP4 deletion (*F*_1,12_ = .649, *P* = .443). In addition, protein levels of Aβ degradation enzymes NEP and IDE, as well as LRP‐1 (a main transporter for Aβ efflux at the BBB),^33^ were comparable among different groups (Figure 2J,K).

### 3.3 Impaired AQP4 polarization in the hippocampus of WT‐SD mice

**Figure 3:**
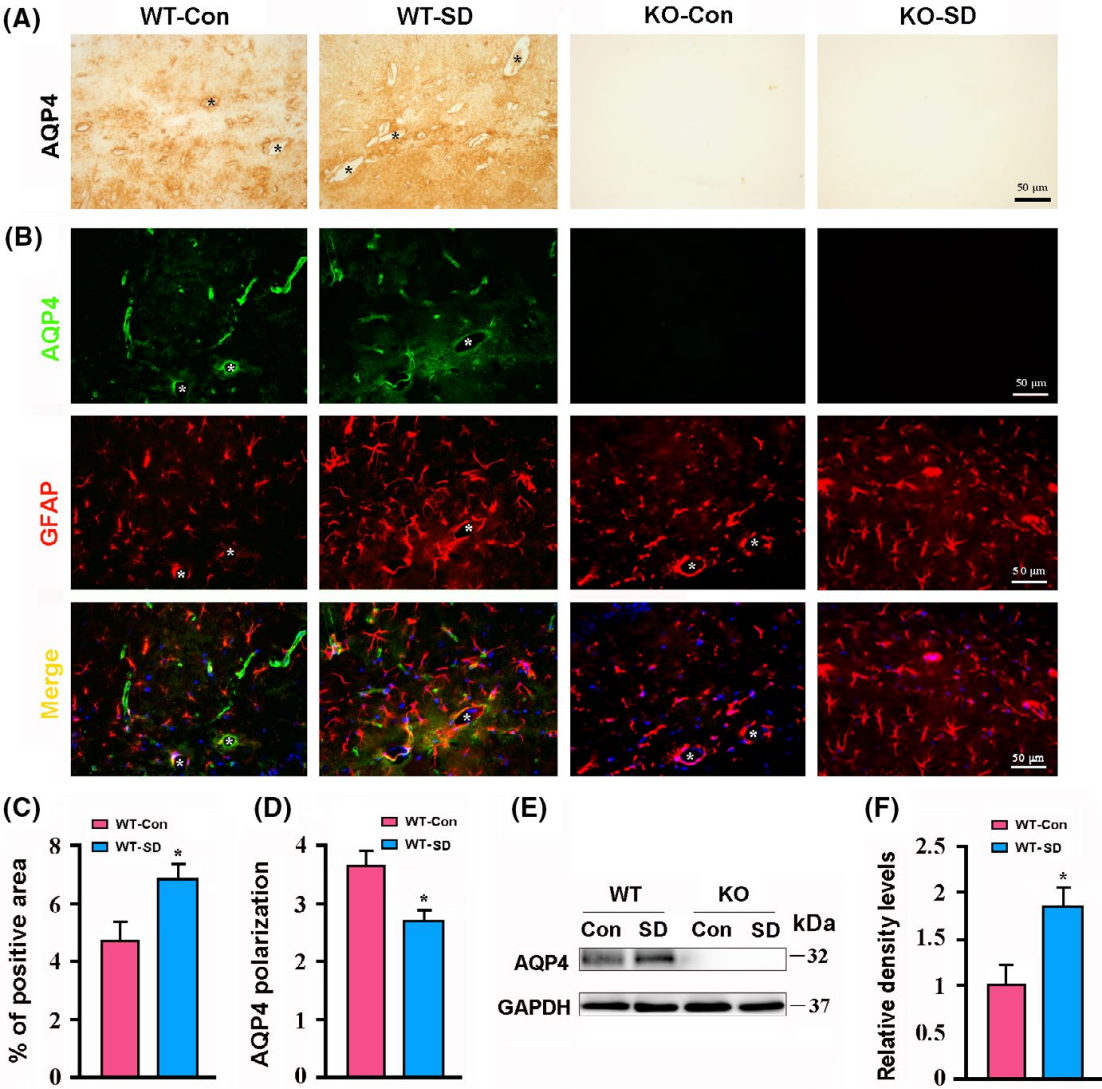
Analysis of aquaporin 4 (AQP4) expression and polarization in the hippocampus of WT and AQP4 KO mice following sleep disruption (SD). A, Representative immunohistochemical images showing expression of AQP4 in the lacunosum moleculare layer of hippocampus. B, Representative images showing coexpression of AQP4 and GFAP in the lacunosum moleculare layer of hippocampus. Note that AQP4 immunoreactivity was mainly around microvessels (stars) in WT‐Con mice. However, this expression pattern was disrupted because AQP4 is abnormally localized to the parenchymal domains. C, Percentage of AQP4‐positive area in the hippocampus. D, AQP4 polarization in the hippocampus. E, F, Representative Western blot bands and densitometry analysis of AQP4 expression from the hippocampal samples. Data represent mean ± SEM from four mice per group. Data were analyzed by Student's *t* test, *P < .05, SD vs Con. KO, knockout; WT, wild‐type

Aquaporin 4 is a water‐selective membrane transport protein expressed in the vascular end‐feet of astrocytes throughout the brain.^34^ Previous studies have demonstrated that the loss of perivascular AQP4 polarization may contribute to glymphatic clearance dysfunction.^14^, ^15^, ^16^, ^17^ We assessed the effect of AQP4 deletion and chronic SD on the expression levels and perivascular polarization of AQP4. Both immunohistochemistry and immunofluorescence revealed that AQP4 expression increased in the hippocampal parenchyma of WT‐SD mice, causing decreases in perivascular AQP4 polarization, compared with WT‐Con mice (both *P* < .05; Figure 3A‐D). Consistently, Western blot revealed that protein levels of AQP4 were up‐regulated in the hippocampus of WT mice after SD (*P* < .05; Figure 3E,F). No AQP4 was detected in brain sections or homogeneous brain samples of AQP4 KO mice (Figure 3A,B,E).

### 3.4 AQP4 deletion increases activation of microglia and neuroinflammation after chronic SD

**Figure 4:**
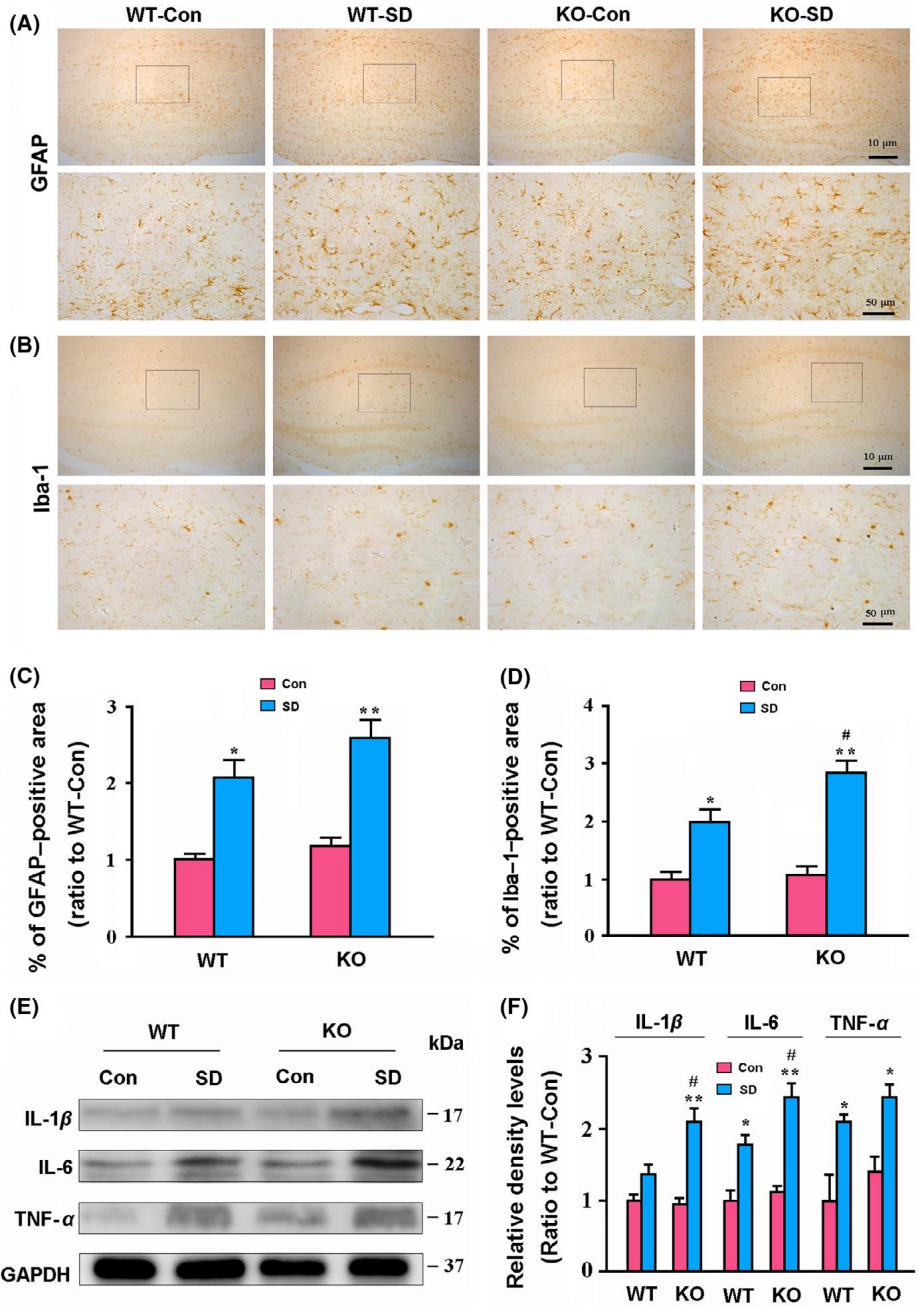
Analysis of glial activation and neuroinflammatory response in the hippocampus of WT and AQP4 KO mice following sleep disruption (SD). A, B, Representative low and high magnification micrographs showing expression and distribution of GFAP‐positive astrocytes (A) and Iba‐1‐positive microglia (B) in the hippocampus. C, D, The percentage of GFAP (C)‐ and Iba‐1 (D)‐positive area in the hippocampus, respectively. E, F, Representative Western blot bands and densitometry analysis of the expression levels of IL‐1β, IL‐6, and TNF‐α from the hippocampal samples. Data represent mean ± SEM from four mice per group and analyzed by the two‐way ANOVA with Tukey's post hoc test. *P < .05, **P < .01, SD vs Con; ^#^ *P* < .05, ^##^ *P* < .01, AQP4 KO vs WT. AQP4, aquaporin 4; KO, knockout; WT, wild‐type

Previous studies, including those from our group, demonstrated that chronic sleep deprivation causes activation of glial cells.^35^, ^36^ Consistently, GFAP‐positive astrocytes and Ibal‐positive microglia were activated in the hippocampus of the both genotype mice after 7‐day SD. AQP4 KO‐SD mice showed more obvious activation of microglia but not astrocytes than WT‐SD mice (Figure 4A,B). Semi‐quantitative analysis further revealed that AQP4 KO‐SD mice had a high percentage of Iba‐1‐positive area (*P* < .05), but not GFAP‐positive area (*P* > .05), when compared to WT‐SD mice (Figure 4C,D). Consistent with increased reactive microgliosis, AQP4 KO‐SD mice had higher hippocampal IL‐1β and IL‐6 expression levels than WT‐SD mice (both *P* < .05; Figure 4E,F).

### 3.5 AQP4 deletion aggravates activation of NLRP3 inflammasomes in the hippocampus after chronic SD

**Figure 5:**
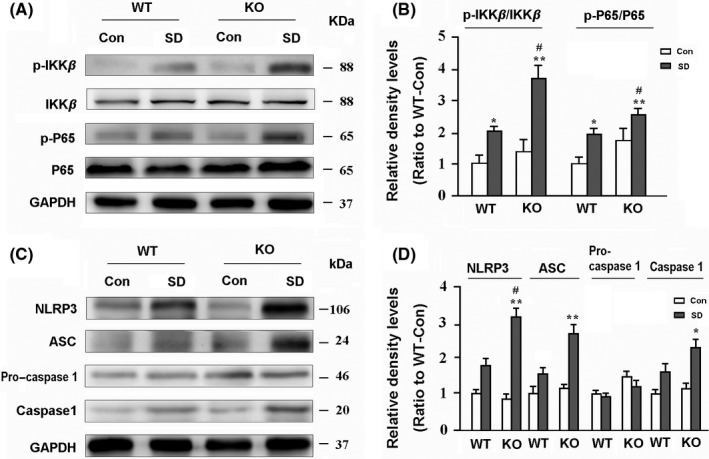
Analysis of activation of NLRP3 inflammasomes in the hippocampus. A, B, Representative Western blot bands and densitometry analysis of the expression levels of the first signal markers including phospho‐P65 (p‐P65), P65, phosphor‐IKKβ (p‐IKKβ), and IKKβ. C, D, Representative Western blot bands and densitometry analysis of the expression levels of the second signal markers including NLRP3, ASC, pro‐caspase 1, and caspase 1 in the hippocampus of mice. Data represent mean ± SEM from four mice per group. The two‐way ANOVA with Tukey's post hoc test. *P < .05, **P < .01, SD vs Con; ^#^ *P* < .05, ^##^ *P* < .01, AQP4 KO vs WT. AQP4, aquaporin 4; KO, knockout; SD, sleep disruption; WT, wild‐type

Accumulating evidence suggests that sleep deprivation evokes activation of NLRP3 inflammasomes in the brain, and triggers overproduction of inflammatory factors, resulting in brain damage.^37^, ^38^, ^39^ Nuclear factor (NF)‐κB transcription signaling pathway, the first signal of NLRP3 activation, plays a critical role in controlling cellular expression of proinflammatory genes.^40^ Western blot results demonstrated that AQP4 deletion enhanced activation of the NF‐κB pathway in the hippocampus of mice after SD, reflected by high ratios of p‐IKKβ/IKKβ and p‐P65/P65 (both *P* < .05; KO‐SD vs WT‐SD; Figure 5A,B). We also investigated several proteins including NLRP3, ASC, pro‐caspase 1, and caspase 1 that are involved in the formation of NLRP3 inflammasomes.^41^ AQP4 KO‐SD mice showed higher levels of NLRP3 and ASC in the hippocampus than WT‐SD mice (both *P* < .05; Figure 5C,D).

### 3.6 AQP4 deletion aggravates synaptic protein loss in the hippocampus after chronic SD

**Figure 6:**
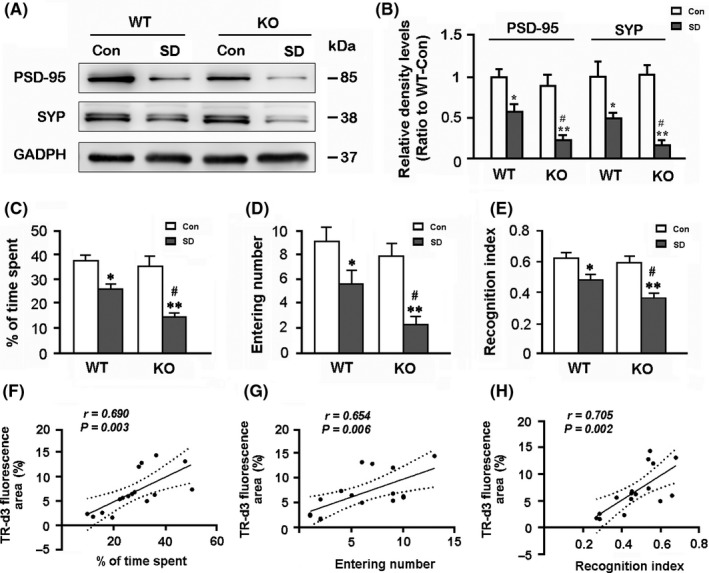
Analysis of synaptic protein expression in the hippocampus, short‐term working memory, and correlation between glymphatic transport and memory‐related parameters. A, B, Representative bands of Western blot and densitometry analysis of PSD‐95 and SYP protein levels in the hippocampal samples. C, The percentage of time spent in the novel arm during the Y‐maze test. D, The number of entries into the novel arm. E, The recognition index (Novel object/Novel object + Familiar object) in the NORT. F‐H, Percentage of cerebrospinal fluid tracer influx into the brain parenchyma partially correlated with short‐term working memory relate indexes including percentage of time spent in the novel arm of the Y‐maze, times of entering into the novel arm, and recognition index of NORT. Data represent mean ± SEM from four mice per group in (B) and 10 mice per group in (C‐E), and analyzed by the two‐way ANOVA with Tukey's post hoc test. *P < .05, **P < .01, SD vs Con; ^#^ *P* < .05, ^##^ *P* < .01, AQP4 KO vs WT. Data in (F‐H) from four mice per group were analyzed by Pearson correlation analysis. AQP4, aquaporin 4; KO, knockout; SD, sleep disruption; WT, wild‐type

Sleep deprivation has a negative effect on synaptic plasticity and transmission, even causing loss of synaptic proteins.^42^, ^43^, ^44^ We observed effects of AQP4 deletion and/or SD on expression of presynaptic and postsynaptic makers SYP and PSD‐95^44^ in the hippocampus. Both AQP4 KO‐SD mice and WT‐SD mice showed decreases in expression levels of SYP and PSD‐95 (*P* < .05, WT‐SD vs WT‐Con; *P* < .01, KO‐SD vs KO‐Con). Moreover, AQP4 deletion further decreased PSD‐95 and SYP levels after chronic SD (both *P* < .05, KO‐SD vs WT‐SD; Figure 6A,B).

### 3.7 AQP4 deletion aggravates mouse memory deficits after chronic SD

Consistent with exacerbated neuroinflammation and synaptic protein loss in the hippocampus, AQP4 deletion magnified short‐term memory due to chronic SD. The Y‐maze test revealed that the percentage of time spent and entry numbers into the NA decreased after SD (*F*_1,36_ = 36.640, *P* < .001; *F*_1,36_ = 26.520, *P* < .001, respectively), but was more apparent in AQP4 KO mice (both *P* < .05; KO‐SD vs WT‐SD; Figure 6C,D). SD also resulted in decreased ability to discriminate between the novel and the familiar objects of the two genotypes (*F*_1,36_ = 47.380, *P* < .001). However, reduction in the recognition index was more significant in AQP4 KO‐SD mice than WT‐SD mice (*P* < .05; Figure 6E).

### 3.8 Correlation between glymphatic dysfunction and short‐term memory deficits after chronic SD

Correlation analysis revealed that the percentages of TR‐d3‐positive areas in the brain had a positive relationship with the percentage of time spent in the novel arm (*r* = .690, *P* = .003), novel arm entrances (*r* = .654, *P* = .006), and recognition index (*r* = .705, *P* = .002; Figure 6F‐H).

## 4 DISCUSSION

Insufficient sleep is very common in modern life, but its damage to the brain cannot be ignored. For examples, Havekes and colleagues reported that even 5 hours of sleep deprivation leads to the loss of dendritic spines of CA1 in mice.^42^ Two or three days of acute sleep deprivation causes synaptic protein loss in the hippocampus.^43^, ^44^ Therefore, it is very important to clarify the molecular mechanism of brain impairments caused by sleep loss.

Recent studies have revealed that sleep facilitates clearance of Aβ and Tau, two key proteins that drive AD pathology, from the brain via an accelerated ISF‐CSF bulk flow.^5^, ^6^ In contrast, following just one night of sleep deprivation, the Aβ burden in the human brain increases.^30^ Chronic sleep deprivation also elevates ISF Aβ levels in the mouse brain.^45^ Holth et al^6^ reported that ISF Tau is regulated by the sleep‐wake cycle, and both ISF Tau in mice and CSF Tau in humans are robustly increased by sleep deprivation. These results suggest that sleep facilities removal of soluble waste proteins from the brain, thus decreases the potential of AD occurrence.

Extracellular Aβ and Tau can be cleared from the brain by various clearance systems, including degradation, ISF bulk flow, and CSF absorption clearance. However, the interaction of clearance mechanisms of these toxic proteins remains unclear. Increasing evidence in the last few years suggests that the glymphatic system could play a crucial role in extracellular Aβ clearance.^10^, ^15^, ^18^ Recent studies have shown that glymphatic transport function is suppressed after sleep deprivation.^7^, ^8^ In agreement with these findings, the present study has revealed that 7‐day SD significantly decreases CSF tracer. Surprisingly, WT‐SD mice show normal Aβ_1‐40_ and Tau protein content in the brain. This phenomenon is likely due to enhanced phagocytosis and degradation of Aβ and Tau proteins by activated astrocytes and microglia. However, absence of AQP4, a glymphatic functional protein, aggravates the damage of glymphatic clearance caused by chronic SD, resulting in significant increases in Aβ and Tau protein levels in the hippocampus. This result suggests that AQP4 has a direct protective effect against accumulation of Aβ and Tau in the brain following chronic SD.

Growing evidence supports that glymphatic clearance is dependent on the polarized localization of AQP4 to perivascular end‐feet. This facilitates the influx of subarachnoid CSF from para‐arterial spaces into the brain interstitium, as well as the subsequent clearance of ISF, via convective bulk flow. Deletion of AQP4, or loss of AQP4 polarity, significantly reduces glymphatic clearance function.^10^, ^14^, ^15^, ^16^, ^17^, ^18^ A recent study reported that chronic sleep deprivation results in decreased efficiency of solute clearance in the perivascular space and loss of AQP4 polarity in the cerebral cortex.^8^ In agreement with this finding, we have confirmed that 1 week of SD causes abnormally increased expression of AQP4 in the brain parenchyma, with associated decreases in CSF tracer influx. These data further suggest that abnormal AQP4 expression is involved in impaired glymphatic clearance after sleep deprivation or insufficiency.

Consistent with previous studies, present results have also revealed that AQP4 deficiency in mice does not change brain Aβ and Tau levels under baseline conditions.^17^, ^18^, ^46^ This could be associated with normal expression levels of enzymes and proteins related to Aβ production and degradation (Figure 2G‐I), as well as the intact meningeal lymphatic draining pathway. This presumption is supported by evidence that AQP4 deletion exacerbates accumulation of soluble Aβ levels in the hippocampus after meningeal lymphatic drainage blocking via ligation of deep cervical lymphatic nodes. 47 Furthermore, we have demonstrated that chronic SD increases BACE1 expression in the hippocampus in WT and AQP4 KO mice. BACE1 has been shown to be a stress‐response protein, which is up‐regulated in various pathological conditions, including oxidative stress, hypoxia, and neuroinflammation.^48^, ^49^ These factors might contribute to SD‐induced BACE1 up‐regulation in the brain, although the underlying mechanism remains elusive.

In addition, a previous study indicated that a facilitating role of sleep on brain macromolecular removal is associated with enhanced convective exchange of cerebrospinal fluid with ISF due to an increase in the brain ISF space.^5^ AQP4 KO mice show a slight increase in ISF extracellular space and brain water content in the baseline condition.^50^ Further investigation is needed to determine whether chronic SD disrupts sleep‐wake cycle‐dependent changes of brain ISF volume and the consequence of AQP4 deletion in this process. The biological rhythms and sleep loss of the two genotypes of mice during the SD process also need to be precisely defined, although AQP4 deletion does not significantly affect activity rhythm after chronic SD. Furthermore, anesthesia has been shown to strongly impact glymphatic flux.^5^ In this study, we evaluated CSF tracer influx of mice after anesthesia and found that AQP4 KO exacerbates glymphatic pathway dysfunction after chronic SD. However, the possibility that changes of glymphatic transport in response to anesthesia are different within individual groups could not be excluded.

Previous studies suggest that chronic sleep deprivation leads to the activation of astrocytes and microglia,^35^, ^36^ which is a main resource for secretion of inflammatory cytokines in the brain. Consistently, in the present study, we have found that both astrocytes and microglia are activated in the hippocampus after chronic SD. Moreover, we have demonstrated that deletion of AQP4 increases activation of hippocampal microglia but not astrocytes in SD mice. Indeed, AQP4 deletion‐attenuated astrocyte activation has been observed in mouse models of experimental autoimmune encephalomyelitis, AD and PD, and in astrocyte culture exposed to Aβ or glutamate.^18^, ^51^, ^52^, ^53^ Particularly, previous studies have indicated that AQP4 has an intrinsic proinflammatory role during astrocyte activation. Reduced IL‐6 and TNF‐α secretion occurs in AQP4 null astrocyte cultures exposed to LPS.^54^ In the present study, high levels of IL‐1β and IL‐6, with activation of NLRP3 inflammasomes, are observed in the hippocampus of AQP4 KO‐SD mice, compared to those in WT‐SD mice. This is possibly due to increased activation of microglia cells. More recent studies have proposed that sleep loss improves activated microglial cells ability to engulf adjacent synaptic structures.^36^ Therefore, aside from toxicity of accumulated Aβ and phosphorylated Tau protein, increased microglial activation and neuroinflammation contribute to synaptic protein loss in the hippocampus in AQP4 KO‐SD mice.

It should also be noted that AQP4 exhibits a highly polarized expression at basolateral plasma membranes of ependymal cells and astrocytic membranes facing the pia, in addition to perivascular end‐feet of astrocytes.^55^ There is sporadic occurrence of obstructive hydrocephalus in a small subpopulation (9.6%) of newborn AQP4 KO mice, resulting in ventricular enlargement, progressive encephalomegaly, and mortality by postnatal week 6.^56^ Adult AQP4 KO mice show decreased CSF production and mildly increased brain water content compared with WT mice.^57^ AQP4 also functionally relates to other astrocyte proteins, including glutamate transport‐1,^58^, ^59^ connexin‐43,^60^ and connexin‐30.^57^ Therefore, besides suppression of glymphatic transport, alterations in blood‐CSF barrier function and these astrocyte functional regulators may also be involved in accumulation of toxic proteins and neuroinflammation observed in AQP4 KO‐SD mice. Further studies are needed to confirm this possibility.

In summary, the present results reveal that AQP4 deletion in SD mice increases glymphatic transport impairment and accumulation of brain Aβ and Tau proteins. AQP4 absence also aggravates chronic SD‐induced activation of microglia, neuroinflammation, and synaptic protein loss in the hippocampus, as well as exacerbating hippocampus‐related memory deficits. These results suggest that AQP4‐mediated glymphatic clearance ameliorates brain impairments caused by abnormal accumulation of metabolic wastes following chronic SD. Despite an intrinsic proinflammatory role during astrocyte activation, AQP4 might serve as a promising functional regulator of the glymphatic system for the treatment of sleep‐related disorders.

## CONFLICT OF INTEREST

The authors declare no conflict of interest.
